# *cis*-Dichloridotetra­kis­(trimethyl­phosphane-κ*P*)ruthenium(II) benzene disolvate

**DOI:** 10.1107/S1600536810049706

**Published:** 2010-12-04

**Authors:** Chen Fu, Ting Bin Wen

**Affiliations:** a Department of Chemistry, College of Chemistry and Chemical Engineering, Xiamen University, Xiamen 361005, Fujian, People’s Republic of China

## Abstract

The title compound, *cis*-[RuCl_2_(C_3_H_9_P)_4_]·2C_6_H_6_, contains a complex mol­ecule with a crystallographic mirror plane passing through the Ru^II^ atom, the two *cis*-disposed Cl ligands and two P atoms of the two *cis*-disposed P(CH_3_)_3_ ligands. The Ru^II^ atom adopts a distorted octa­hedral RuCl_2_P_4_ coordination geometry with the two *trans*-disposed P atoms occupying the axial positions. The packing of the structure is accomplished through non-classical C—H⋯Cl hydrogen bonds between the benzene solvent mol­ecule and one of the Cl ligands.

## Related literature

For general background to *trans*-[RuCl_2_(P(CH_3_)_3_)_4_], see: Csok *et al.* (2007 ▶); Gotzig *et al.* (1985 ▶); Hartwig *et al.* (1991 ▶); Hirano *et al.* (2010 ▶); Kohlmann & Werner (1993 ▶). For a related structure, see: Joo *et al.* (1994 ▶).
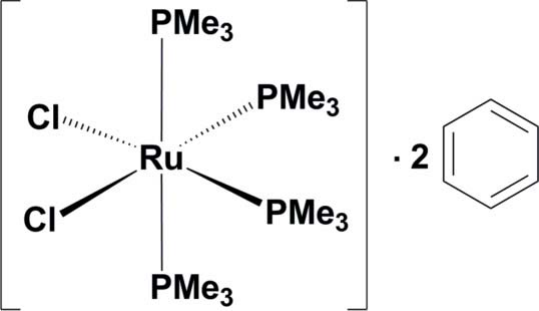


## Experimental

### 

#### Crystal data

[RuCl_2_(C_3_H_9_P)_4_]·2C_6_H_6_
*M* *_r_* = 632.47
Orthorhombic, 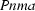

*a* = 17.6243 (15) Å
*b* = 18.1889 (19) Å
*c* = 9.4610 (11) Å
*V* = 3032.9 (5) Å^3^
*Z* = 4
Mo *K*α radiation
μ = 0.92 mm^−1^
*T* = 173 K
0.18 × 0.12 × 0.06 mm

#### Data collection

Oxford Diffraction Gemini S Ultra diffractometer
Absorption correction: multi-scan (*CrysAlis RED*; Oxford Diffraction, 2008 ▶) *T*_min_ = 0.949, *T*_max_ = 1.000
14352 measured reflections
3550 independent reflections
1653 reflections with *I* > 2σ(*I*)
*R*_int_ = 0.148

#### Refinement

*R*[*F* ^2^ > 2σ(*F* ^2^)] = 0.058
*wR*(*F* ^2^) = 0.081
*S* = 0.82
3550 reflections
139 parameters
H-atom parameters constrained
Δρ_max_ = 0.97 e Å^−3^
Δρ_min_ = −0.57 e Å^−3^

### 

Data collection: *CrysAlis CCD* (Oxford Diffraction, 2008 ▶); cell refinement: *CrysAlis RED* (Oxford Diffraction, 2008 ▶); data reduction: *CrysAlis RED*; program(s) used to solve structure: *SHELXTL* (Sheldrick, 2008 ▶); program(s) used to refine structure: *SHELXTL*; molecular graphics: *SHELXTL*; software used to prepare material for publication: *SHELXTL*.

## Supplementary Material

Crystal structure: contains datablocks I, global. DOI: 10.1107/S1600536810049706/wm2431sup1.cif

Structure factors: contains datablocks I. DOI: 10.1107/S1600536810049706/wm2431Isup2.hkl

Additional supplementary materials:  crystallographic information; 3D view; checkCIF report

## Figures and Tables

**Table 1:** Selected bond lengths (Å)

Ru1—P1	2.2690 (19)
Ru1—P2	2.297 (2)
Ru1—P3	2.3819 (14)
Ru1—Cl1	2.479 (2)
Ru1—Cl2	2.5038 (19)

**Table 2:** Hydrogen-bond geometry (Å, °)

*D*—H⋯*A*	*D*—H	H⋯*A*	*D*⋯*A*	*D*—H⋯*A*
C4*S*—H4*SA*⋯Cl2	0.93	2.83	3.710 (5)	159

**Fig. 1.**
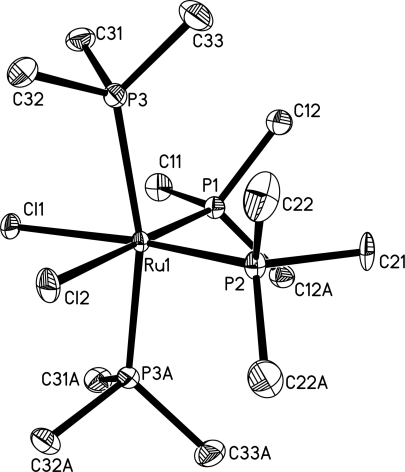
The coordination of the RuII atom in the structure of cis-[RuCl2(P(CH3)3)4] with the atom labelling and displacement ellipsoids drawn at the 30% probability level. H atoms have been omitted for clarity. [Symmetry code: (A) x, -y+1/2, z.]

**Fig. 2.**
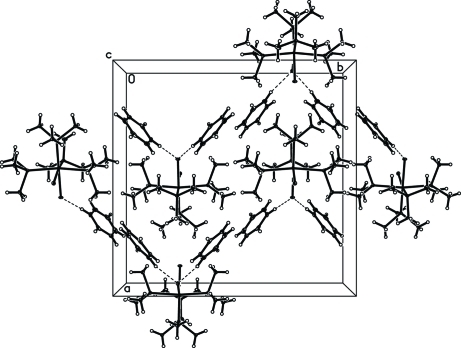
The packing diagram of the structure viewed down the c axis, showing the non-classical C—H···Cl hydrogen bonds between the benzene solvent molecule and one of the Cl ligands of the complex molecule.

## supplementary crystallographic information

### Comment

The ruthenium(II) complex *trans*-[RuCl_2_(PMe_3_)_4_], which can be readily prepared from the reaction of RuCl_2_(PPh_3_)_3_ with PMe_3_ in hexane at room temperature (Gotzig *et al.*, 1985), has proved to be a useful precursor for a wide variety of ruthenium compounds (Hartwig *et al.*, 1991; Kohlmann & Werner, 1993; Csok *et al.*, 2007; Hirano *et al.*, 2010). However, its geometrical isomer, *cis*-[RuCl_2_(PMe_3_)_4_], has not been reported yet. During our preparation of ruthenium compounds with phosphine ligands using *trans*-[RuCl_2_(PMe_3_)_4_] as the starting material, we found that the *trans*-isomer slowly isomerizes to the *cis*-isomer, the structure of which we report here as the benzene disolvate.

As shown in Fig.1, the structure of the title complex possesses a crystallographic mirror plane passing through the Ru^II^ atom, the two *cis*-disposed Cl ligands and the two P atoms as well as two C atoms of the two *cis*-disposed PMe_3_ ligands. Thus the asymmetric unit of the structure contains half of a molecule. The Ru^II^ atom adopts a distorted octahedral geometry with the two *trans*-disposed P atoms occupying the axial positions. The bond lengths of the two axial Ru—P bonds (2.3819 (14) Å), which are actually image-related, are slightly longer than those of the two equatorial Ru—P bonds which are *trans* to the Cl ligands (2.2690 (19) and 2.297 (2) Å, respectively). The two Ru—Cl bond lengths are 2.479 (2) and 2.5038 (19) Å, respectively, while the Cl(1)—Ru(1)—Cl(2) bond angle is 86.20 (7)°. These geometric values are similar to those reported for the only example of a sructurally characterized monodentate tetrakis(phosphine)-*cis*-dichlorido-ruthenium(II) complex, *viz*. [*cis*-RuCl_2_(PTA)_4_] (PTA = 1,3,5-triaza-7-phospha-adamantane-κP); 2.488 (2) and 2.503 (2) Å, 84.2 (1) °; Joo *et al.*, 1994).

The packing of the structure (Fig. 2) is accomplished through non-classical C—H···Cl hydrogen bonds between the benzene solvent molecule and one of the Cl ligands (Table 2).

### Experimental

Route A: To a solution of RuCl_2_(PPh_3_)_3_ (0.25 g, 0.24 mmol) in toluene (8 ml) under nitrogen atmosphere was added PMe_3_ (0.27 ml, 2.5 mmol) and the resulting yellow solution was refluxed for 20 h. The solvent was removed under vacuum, and the solid residue was washed with n-hexane, and dried under vacuum to afford a white solid. Yield: 90 mg, 80%. Route B: A solution of *trans*-[RuCl_2_(PMe_3_)_4_] (100 mg, 0.21 mmol) in benzene (5 ml) was refluxed for 12 h under nitrogen atmosphere. After the solution was cooled to ambient temperature, a white solid was precipitated, which was collected by filtration, washed with n-hexane, and dried under vacuum. Yield: 72 mg, 72%. Crystals suitable for X-ray analysis were obtained from standing a solution of *trans*-[RuCl_2_(PMe_3_)_4_] in benzene at room temperature for 2 weeks.

### Refinement

The benzene solvent molecule was treated as a rigid body. All non-hydrogen atoms were refined anisotropically. The hydrogen atoms were positioned geometrically (C—H = 0.96 or 0.93 Å for methyl or phenyl H atoms, respectively) and were included in the refinement in the riding model approximation. The displacement parameters of methyl H atoms were set to 1.5*U*_eq_(C), while those of the phenyl H atoms were set to 1.2*U*_eq_(C). In the final Fourier map the highest peak is 0.04 Å from atom Ru1 and the deepest hole is 0.71 Å from atom Ru1.

### Crystal data

**Table:**

[RuCl_2_(C_3_H_9_P)_4_]·2C_6_H_6_	*F*(000) = 1320
*M**_r_* = 632.47	*D*_x_ = 1.385 Mg m^−^^3^
Orthorhombic, *P**n**m**a*	Mo *K*α radiation, λ = 0.7107 Å
Hall symbol: -P 2ac 2n	Cell parameters from 1376 reflections
*a* = 17.6243 (15) Å	θ = 2.3–28.9°
*b* = 18.1889 (19) Å	µ = 0.92 mm^−^^1^
*c* = 9.4610 (11) Å	*T* = 173 K
*V* = 3032.9 (5) Å^3^	Block, colorless
*Z* = 4	0.18 × 0.12 × 0.06 mm

### Data collection

**Table:**

Oxford Diffraction Gemini S Ultra diffractometer	3550 independent reflections
Radiation source: Enhance (Mo) X-ray Source	1653 reflections with *I* > 2σ(*I*)
graphite	*R*_int_ = 0.148
Detector resolution: 16.1930 pixels mm^-1^	θ_max_ = 27.5°, θ_min_ = 2.3°
ω scans	*h* = −22→21
Absorption correction: multi-scan (*CrysAlis RED*; Oxford Diffraction, 2008)	*k* = −23→22
*T*_min_ = 0.949, *T*_max_ = 1.000	*l* = −10→12
14352 measured reflections	

### Refinement

**Table:**

Refinement on *F*^2^	Primary atom site location: structure-invariant direct methods
Least-squares matrix: full	Secondary atom site location: difference Fourier map
*R*[*F*^2^ > 2σ(*F*^2^)] = 0.058	Hydrogen site location: inferred from neighbouring sites
*wR*(*F*^2^) = 0.081	H-atom parameters constrained
*S* = 0.82	*w* = 1/[σ^2^(*F*_o_^2^) + (0.0104*P*)^2^] where *P* = (*F*_o_^2^ + 2*F*_c_^2^)/3
3550 reflections	(Δ/σ)_max_ = 0.003
139 parameters	Δρ_max_ = 0.97 e Å^−^^3^
0 restraints	Δρ_min_ = −0.57 e Å^−^^3^

### Special details

**Table:**

Geometry. All e.s.d.'s (except the e.s.d. in the dihedral angle between two l.s. planes) are estimated using the full covariance matrix. The cell e.s.d.'s are taken into account individually in the estimation of e.s.d.'s in distances, angles and torsion angles; correlations between e.s.d.'s in cell parameters are only used when they are defined by crystal symmetry. An approximate (isotropic) treatment of cell e.s.d.'s is used for estimating e.s.d.'s involving l.s. planes.
Refinement. Refinement of *F*^2^ against ALL reflections. The weighted *R*-factor *wR* and goodness of fit *S* are based on *F*^2^, conventional *R*-factors *R* are based on *F*, with *F* set to zero for negative *F*^2^. The threshold expression of *F*^2^ > σ(*F*^2^) is used only for calculating *R*-factors(gt) *etc*. and is not relevant to the choice of reflections for refinement. *R*-factors based on *F*^2^ are statistically about twice as large as those based on *F*, and *R*- factors based on ALL data will be even larger.

### Fractional atomic coordinates and isotropic or equivalent isotropic displacement parameters (Å^2^)

**Table:**

	*x*	*y*	*z*	*U*_iso_*/*U*_eq_	
Ru1	0.54358 (3)	0.2500	0.30360 (7)	0.01803 (17)	
Cl1	0.47805 (10)	0.2500	0.0718 (2)	0.0308 (6)	
Cl2	0.41377 (9)	0.2500	0.4111 (2)	0.0347 (6)	
P1	0.66211 (10)	0.2500	0.2100 (2)	0.0221 (5)	
P2	0.57932 (10)	0.2500	0.5371 (2)	0.0233 (6)	
P3	0.53321 (8)	0.37973 (7)	0.27518 (17)	0.0273 (4)	
C11	0.6660 (4)	0.2500	0.0209 (8)	0.032 (2)	
H11A	0.7178	0.2500	−0.0104	0.049*	
H11B	0.6408	0.2069	−0.0142	0.049*	
C12	0.7266 (2)	0.3256 (2)	0.2488 (6)	0.0303 (17)	
H12A	0.7740	0.3175	0.2015	0.045*	
H12B	0.7350	0.3282	0.3489	0.045*	
H12C	0.7047	0.3709	0.2165	0.045*	
C21	0.6769 (3)	0.2500	0.5976 (8)	0.036 (2)	
H21A	0.6785	0.2500	0.6990	0.055*	
H21B	0.7020	0.2069	0.5623	0.055*	
C22	0.5445 (3)	0.3255 (3)	0.6447 (6)	0.057 (2)	
H22A	0.5627	0.3200	0.7397	0.086*	
H22B	0.4900	0.3252	0.6447	0.086*	
H22C	0.5624	0.3713	0.6065	0.086*	
C31	0.5557 (3)	0.4150 (3)	0.1015 (6)	0.0368 (16)	
H31A	0.5499	0.4675	0.1010	0.055*	
H31B	0.5221	0.3936	0.0331	0.055*	
H31C	0.6072	0.4027	0.0781	0.055*	
C32	0.4370 (2)	0.4140 (3)	0.2916 (7)	0.0447 (18)	
H32A	0.4369	0.4664	0.2789	0.067*	
H32B	0.4177	0.4023	0.3837	0.067*	
H32C	0.4056	0.3916	0.2208	0.067*	
C33	0.5837 (3)	0.4488 (3)	0.3798 (6)	0.046 (2)	
H33A	0.5700	0.4971	0.3476	0.069*	
H33B	0.6374	0.4420	0.3690	0.069*	
H33C	0.5703	0.4436	0.4776	0.069*	
C1S	0.2535 (3)	0.4577 (2)	0.8727 (8)	0.069 (3)	
H1SA	0.2273	0.4919	0.9273	0.082*	
C2S	0.3127 (4)	0.4177 (4)	0.9316 (4)	0.073 (3)	
H2SA	0.3263	0.4251	1.0255	0.088*	
C3S	0.3518 (2)	0.3667 (3)	0.8500 (8)	0.068 (3)	
H3SA	0.3915	0.3399	0.8894	0.081*	
C4S	0.3316 (3)	0.3557 (2)	0.7096 (8)	0.059 (2)	
H4SA	0.3578	0.3215	0.6550	0.071*	
C5S	0.2724 (3)	0.3957 (3)	0.6507 (4)	0.059 (2)	
H5SA	0.2589	0.3883	0.5567	0.071*	
C6S	0.23327 (19)	0.4467 (3)	0.7323 (8)	0.064 (3)	
H6SA	0.1936	0.4735	0.6929	0.076*	

### Atomic displacement parameters (Å^2^)

**Table:**

	*U*^11^	*U*^22^	*U*^33^	*U*^12^	*U*^13^	*U*^23^
Ru1	0.0145 (3)	0.0200 (3)	0.0196 (4)	0.000	−0.0017 (4)	0.000
Cl1	0.0333 (12)	0.0318 (13)	0.0275 (14)	0.000	−0.0146 (10)	0.000
Cl2	0.0154 (11)	0.0489 (14)	0.0398 (15)	0.000	−0.0001 (10)	0.000
P1	0.0212 (10)	0.0254 (12)	0.0197 (14)	0.000	0.0015 (11)	0.000
P2	0.0181 (11)	0.0330 (14)	0.0187 (14)	0.000	0.0012 (10)	0.000
P3	0.0230 (8)	0.0249 (8)	0.0339 (11)	0.0015 (7)	−0.0029 (8)	−0.0031 (8)
C11	0.029 (5)	0.036 (6)	0.032 (6)	0.000	0.012 (4)	0.000
C12	0.026 (3)	0.029 (3)	0.036 (5)	0.002 (3)	0.003 (3)	0.000 (3)
C21	0.023 (5)	0.073 (7)	0.013 (5)	0.000	−0.004 (4)	0.000
C22	0.059 (4)	0.081 (5)	0.032 (4)	0.027 (4)	−0.006 (4)	−0.018 (4)
C31	0.048 (4)	0.027 (3)	0.035 (4)	−0.003 (3)	−0.002 (3)	0.008 (3)
C32	0.039 (4)	0.031 (3)	0.064 (5)	0.009 (3)	−0.008 (4)	−0.001 (4)
C33	0.039 (4)	0.033 (4)	0.066 (6)	−0.007 (3)	0.003 (3)	−0.013 (4)
C1S	0.085 (6)	0.053 (6)	0.067 (7)	−0.029 (5)	0.050 (5)	−0.019 (6)
C2S	0.099 (7)	0.091 (8)	0.029 (5)	−0.066 (5)	−0.019 (5)	0.026 (6)
C3S	0.039 (4)	0.063 (6)	0.101 (9)	−0.009 (4)	−0.006 (5)	0.041 (6)
C4S	0.056 (5)	0.026 (4)	0.097 (8)	−0.004 (3)	0.049 (5)	−0.009 (5)
C5S	0.087 (6)	0.059 (6)	0.033 (5)	−0.050 (4)	−0.003 (5)	0.007 (5)
C6S	0.035 (4)	0.037 (5)	0.119 (10)	−0.007 (3)	0.010 (5)	0.029 (6)

### Geometric parameters (Å, °)

**Table:**

Ru1—P1	2.2690 (19)	C22—H22B	0.9600
Ru1—P2	2.297 (2)	C22—H22C	0.9600
Ru1—P3^i^	2.3819 (14)	C31—H31A	0.9600
Ru1—P3	2.3819 (14)	C31—H31B	0.9600
Ru1—Cl1	2.479 (2)	C31—H31C	0.9600
Ru1—Cl2	2.5038 (19)	C32—H32A	0.9600
P1—C11	1.790 (8)	C32—H32B	0.9600
P1—C12	1.821 (4)	C32—H32C	0.9600
P1—C12^i^	1.821 (4)	C33—H33A	0.9600
P2—C21	1.812 (6)	C33—H33B	0.9600
P2—C22	1.816 (5)	C33—H33C	0.9600
P2—C22^i^	1.816 (5)	C1S—C2S	1.3900
P3—C31	1.808 (5)	C1S—C6S	1.3900
P3—C32	1.813 (4)	C1S—H1SA	0.9300
P3—C33	1.831 (5)	C2S—C3S	1.3900
C11—H11A	0.9600	C2S—H2SA	0.9300
C11—H11B	0.9598	C3S—C4S	1.3900
C12—H12A	0.9600	C3S—H3SA	0.9300
C12—H12B	0.9600	C4S—C5S	1.3900
C12—H12C	0.9600	C4S—H4SA	0.9300
C21—H21A	0.9600	C5S—C6S	1.3900
C21—H21B	0.9599	C5S—H5SA	0.9300
C22—H22A	0.9600	C6S—H6SA	0.9300
			
P1—Ru1—P2	97.07 (8)	H21A—C21—H21B	109.5
P1—Ru1—P3^i^	91.52 (4)	P2—C22—H22A	109.5
P2—Ru1—P3^i^	97.45 (4)	P2—C22—H22B	109.5
P1—Ru1—P3	91.52 (4)	H22A—C22—H22B	109.5
P2—Ru1—P3	97.45 (4)	P2—C22—H22C	109.5
P3^i^—Ru1—P3	164.31 (8)	H22A—C22—H22C	109.5
P1—Ru1—Cl1	94.79 (8)	H22B—C22—H22C	109.5
P2—Ru1—Cl1	168.14 (7)	P3—C31—H31A	109.5
P3^i^—Ru1—Cl1	82.20 (4)	P3—C31—H31B	109.5
P3—Ru1—Cl1	82.20 (4)	H31A—C31—H31B	109.5
P1—Ru1—Cl2	179.01 (9)	P3—C31—H31C	109.5
P2—Ru1—Cl2	81.94 (7)	H31A—C31—H31C	109.5
P3^i^—Ru1—Cl2	88.61 (4)	H31B—C31—H31C	109.5
P3—Ru1—Cl2	88.61 (4)	P3—C32—H32A	109.5
Cl1—Ru1—Cl2	86.20 (7)	P3—C32—H32B	109.5
C11—P1—C12	100.2 (2)	H32A—C32—H32B	109.5
C11—P1—C12^i^	100.2 (2)	P3—C32—H32C	109.5
C12—P1—C12^i^	98.0 (3)	H32A—C32—H32C	109.5
C11—P1—Ru1	115.2 (2)	H32B—C32—H32C	109.5
C12—P1—Ru1	119.71 (17)	P3—C33—H33A	109.5
C12^i^—P1—Ru1	119.71 (17)	P3—C33—H33B	109.5
C21—P2—C22	98.3 (2)	H33A—C33—H33B	109.5
C21—P2—C22^i^	98.3 (2)	P3—C33—H33C	109.5
C22—P2—C22^i^	98.3 (4)	H33A—C33—H33C	109.5
C21—P2—Ru1	124.3 (3)	H33B—C33—H33C	109.5
C22—P2—Ru1	116.5 (2)	C2S—C1S—C6S	120.0
C22^i^—P2—Ru1	116.5 (2)	C2S—C1S—H1SA	120.0
C31—P3—C32	99.3 (3)	C6S—C1S—H1SA	120.0
C31—P3—C33	98.1 (3)	C3S—C2S—C1S	120.0
C32—P3—C33	99.9 (2)	C3S—C2S—H2SA	120.0
C31—P3—Ru1	115.93 (18)	C1S—C2S—H2SA	120.0
C32—P3—Ru1	113.76 (17)	C2S—C3S—C4S	120.0
C33—P3—Ru1	125.57 (19)	C2S—C3S—H3SA	120.0
P1—C11—H11A	110.1	C4S—C3S—H3SA	120.0
P1—C11—H11B	109.1	C3S—C4S—C5S	120.0
H11A—C11—H11B	109.5	C3S—C4S—H4SA	120.0
P1—C12—H12A	109.5	C5S—C4S—H4SA	120.0
P1—C12—H12B	109.5	C6S—C5S—C4S	120.0
H12A—C12—H12B	109.5	C6S—C5S—H5SA	120.0
P1—C12—H12C	109.5	C4S—C5S—H5SA	120.0
H12A—C12—H12C	109.5	C5S—C6S—C1S	120.0
H12B—C12—H12C	109.5	C5S—C6S—H6SA	120.0
P2—C21—H21A	110.1	C1S—C6S—H6SA	120.0
P2—C21—H21B	109.1		

Symmetry codes: (i) *x*, −*y*+1/2, *z*.

### Hydrogen-bond geometry (Å, °)

**Table:**

*D*—H···*A*	*D*—H	H···*A*	*D*···*A*	*D*—H···*A*
C4S—H4SA···Cl2i	0.93	2.83	3.710 (5)	159.

Symmetry codes: i.
